# Photon-counting CT-guided bone biopsy with real-time bone marrow edema mapping

**DOI:** 10.1186/s41747-026-00690-6

**Published:** 2026-03-16

**Authors:** B. Alvarez de Sierra Garcia, C. Urtasun-Iriarte, P. Nieto, A. Alonso Burgos

**Affiliations:** Department of Radiology, Clínica Universidad de Navarra, Madrid, Spain

**Keywords:** Bone marrow, Bone diseases, Image-guided biopsy, Oedema, Tomography (x-ray computed).

## Abstract

**Objective:**

Computed tomography-guided biopsies are needed to diagnose bone lesions, but can sometimes be challenging. We evaluated the feasibility and usefulness of photon-counting computed tomography (PCCT)-guided bone biopsies, focusing on real-time bone marrow oedema (BME) mapping to optimise diagnostic yield.

**Materials and methods:**

This retrospective single-centre study included procedures performed from September 2024 to May 2025 using a first-generation dual-source PCCT scanner with Quantum HD mode. Ten consecutive patients underwent PCCT-guided bone biopsy with real-time BME reconstructions. The reference standard was established using histopathology or microbiological confirmation when available; clinical and ≥ 3-month radiologic follow-up for nondiagnostic or discordant results. Statistical analysis included descriptive statistics, independent unpaired *t*-tests, and correlation analysis (SPSS v22.0, RStudio).

**Results:**

Ten patients, five women and five men, aged 60.5 ± 13.5 years (mean ± standard deviation), were included in the final analysis. The overall diagnostic yield was 70% (7/10), with a diagnostic accuracy of 87.5% (7/8) for cases with a definitive reference standard. Final diagnoses comprised tumour bone metastases (*n* = 7, 70%), bone osteomyelitis (*n* = 1, 10%), and bone marrow deposition disease (*n* = 2, 20%). Mean radiation dose (dose-length product) was 644.5 ± 112.1 mGy·cm. Monoenergetic 70-keV imaging showed significant differences between mean HU values of lytic (42.6) and sclerotic lesions (476.2) (*p* = 0.009), with a strong negative correlation between lesion morphology (sclerotic *versus* lytic) and monoenergetic 70-keV attenuation values (*r* = -0.84; *p* = 0.002).

**Conclusion:**

PCCT-guided bone biopsy with real-time BME mapping proved feasible and showed encouraging diagnostic performance in this small exploratory cohort. Larger validation studies are needed.

**Relevance statement:**

By combining monoenergetic images and BME mapping, PCCT-guided bone biopsy improves lesion visualisation, operator confidence, procedural efficiency, and overall safety for diagnostic tissue sampling and active disease targeting.

**Key Points:**

- Accurate targeting of active disease within complex bone lesions during CT-guided biopsy remains challenging sometimes.
- PCCT-guided biopsy with real-time BME mapping can enhance lesion targeting and procedural efficiency.
- PCCT-guided biopsy may improve safety, diagnostic accuracy, and operator confidence.

**Graphical Abstract:**

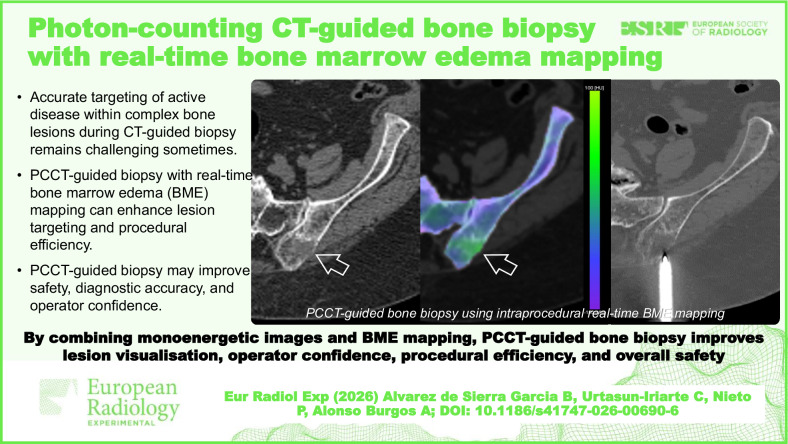

## Background

Photon-counting computed tomography (PCCT), first approved for clinical use by the USA Food and Drug Administration in 2021 [1], has marked a milestone in 21st-century radiology by incorporating a new acquisition technology. PCCT uses semiconductor-based detectors that directly convert x-ray photons into electrical signals, allowing for precise energy discrimination and the acquisition of pure multienergy data in a single scan. This contrasts with conventional spectral energy-integrating computed tomography (CT) detectors, which rely on scintillators to convert x-ray photons into visible light [2, 3]. PCCT equipment also eliminates interpixel septa within detectors, enabling ultrahigh (< 0.2 mm) spatial resolution acquisitions. In combination with energy thresholding technology, which removes electronic noise, this technology improves signal-to-noise ratios, increases radiation-use efficiency, and reduces dose [4].

Conventional CT-guided bone biopsy relies on anatomical landmarks and structural features (*e.g*., cortical destruction) to localise the target point. This approach, whilst adequate for some lesions, presents significant limitations when approaching complex osseous pathologies, particularly sclerotic or mixed lesions, where identifying viable diagnostic tissue remains challenging [5]. Traditional CT-guidance fails to differentiate between metabolically active regions and inactive or necrotic tissue, potentially leading to non-diagnostic sampling [5, 6]. Even though other imaging techniques allow it (magnetic resonance imaging or positron emission tomography-guided biopsy), they are not always available, technically possible or economically feasible.

Pathophysiologically, bone marrow oedema (BME) reflects the marrow’s response to a spectrum of insults, ranging from nonspecific reactive changes—such as trauma, microfractures, or transient vascular congestion—to active pathology, including infectious, inflammatory, and neoplastic conditions. In these scenarios, BME can be precisely assessed by spectral CT imaging [7–9]. Newly introduced PCCT pure multi-energy acquisition allows for real-time virtual non-calcium (VNCa) reconstructions by suppressing calcium attenuation whilst enhancing the visualisation of bone marrow, generating a colour-coded BME map that can be used to assess intramedullary pathologies [10, 11]. This guidance tool might potentially increase diagnostic precision, reduce procedure time, and boost the operator’s targeting confidence. However, real-time mapping of BME during the biopsy itself remains an unexplored frontier.

This study aims to evaluate the clinical usefulness and feasibility of PCCT-guided bone biopsies, specifically by integrating real-time BME mapping via VNCa reconstructions to optimise diagnostic yield and procedural efficiency.

## Materials and methods

### Study design

This retrospective study received approval from the institutional review board (CEIC 2025.016). From September 2024 to May 2025, a total of ten consecutive patients were referred to the Radiology Department to undergo PCCT-guided bone biopsy of suspected bone lesions identified on prior imaging techniques. Informed consent for undergoing the procedure was obtained from all participants. Patients were included if lesions were accessible for safe intervention, tolerated positioning, and had no bleeding disorders, were not taking anticoagulants, and had no active infections. Only one lesion per patient was included for analysis, and all outcomes (diagnostic yield, diagnostic accuracy, imaging metrics, and radiation dose) were calculated on a per-patient basis.

### **PCCT imaging**

The bone biopsies were performed using a first-generation dual-source PCCT scanner (NAEOTOM Alpha, Siemens Healthineers). Scans were conducted in Quantum HD scan mode with a collimation of 96 × 0.2 mm. The system generated spectral imaging results, including single-photon processing, with standard reconstructions performed at slice thicknesses as low as 0.4 mm using kernels of up to 80. Additionally, Quantum HD reconstructions achieved a slice thickness of 0.2 mm. Systems A and B operated at different kilovolt peak (kVp) and threshold settings, with a thick tin filter and a tube voltage of 150 kV. The specific kVp configurations included 70/Sn150 kVp and 90/Sn150 kVp. Only the smallest collimation was employed to ensure ultrahigh resolution imaging, and only noncontrast scan protocols were used for this study. Reconstruction techniques included T3D reconstructions at full ultrahigh resolution, as well as additional spectral reconstructions for monoenergetic images, bone marrow analysis, and single-photon processing (Table 1).

**Table 1:** Acquisition and reconstruction parameters

Scanner model	NAETOM alpha		
Source mode	Dual source		
Tube voltage	70/Sn150		
Pitch	0.6		
Collimation	96 × 0.2 mm		
QIR	None*		
Pixel spacing (in *x*- and *y*-axis)	0.5 mm		
Series	Bone	Soft tissue	BME mapping
Slice thickness	0.6 mm	0.6 mm	2 mm
Slice interval	0.4 mm	0.4 mm	2 mm
Reconstruction filter	Br80	Br40	Qr40
Matrix size	768 × 768	512 × 512	512 × 512

*BME* Bone marrow oedema, *QIR* Quantum iterative reconstruction

* To enable BME map processing, data acquisition must be performed in a specific vendor-defined multi-energy mode. This acquisition mode imposes certain technical limitations, such as the unavailability of QIR implementation

These advanced imaging techniques provided detailed visualisation of the suspect bone lesions, facilitating precise CT-guided biopsy procedures. In the evaluation of bone lesions by PCCT prior to biopsy, key parameters were evaluated to characterise the lesions and improve procedure planning. The analysis identified the number and anatomic location of lesions, predominantly in the iliac, sacral and femoral bones. Lesions were morphologically classified as lytic or sclerotic, measured in millimetres. For each lesion, HU values were recorded at a mean energy level of 70 keV, which closely approximates the attenuation characteristics of 120-kVp imaging while providing improved image quality through reduced noise and artefact levels, thereby enhancing assessment of tissue density and composition [12]. In addition, the presence of oedema was assessed on BME maps, suggesting lesion activity. These findings were instrumental not only in characterising the lesions but also in determining optimal puncture sites, thus improving the diagnostic yield of the biopsy procedure.

For intraprocedural bone-marrow analysis, BME maps were generated using a vendor-specific three-material decomposition model (water, fat, and calcium) applied to the spectral dataset. VNCa processing suppressed calcium attenuation to enhance marrow attenuation differences. The colour scale was vendor-calibrated, with yellow-green values representing increased VNCa attenuation (interpreted as pathological oedema) and blue-violet values representing normal marrow. BME maps were reconstructed using a 2-mm slice thickness and 2-mm increment with the Qr40 spectral kernel, whereas standard high-resolution diagnostic series were reconstructed at 0.6 mm (Br80/Br40) and ultra-high-resolution reconstructions at 0.2–0.4 mm. These parameters are now fully aligned with Table 1.

For each procedure, dose accumulation parameters were recorded in detail. The dose-length product (DLP) reflected the sum of all acquisitions performed during the intervention, including the planning scan, incremental checks during needle advancement, and the final post-biopsy control scan. All BME/VNCa reconstructions were derived from the same spectral dataset and did not require additional acquisitions. For each series, the scan range, collimation, and kVp/threshold settings (70/Sn150 or 90/Sn150 kVp) were documented.

### PCCT-guided bone biopsy technique

The bone biopsy was performed using a battery-powered drill system [13]. Initially, a PCCT scan was conducted to identify the bone lesion. Based on a colour-coded scale of the real-time BME map, a qualitative analysis was performed to localise the most relevant area for biopsy. Shades of yellow-green were considered pathological, while shades of blue-violet were considered normal. The skin was marked, the cutaneous plane was sterilised, and local anaesthetic and intravenous analgesia were administered to ensure patient comfort. Access was achieved by contacting the periosteum, after which the drill was engaged to perforate the cortical bone and reach the lesion. The axial device was then replaced by removing the drill and introducing the bone biopsy system. Three passes were made for each lesion, directed towards the region with the most significant oedema. After the biopsy system was removed, a follow-up scan was performed to confirm no complications, utilising reconstructions of the oedema, bone window, and soft tissue.

A single pathologist with a decade of experience in bone lesions analysed both sclerotic and osteolytic samples.

### Statistical analysis

The statistical analyses were conducted using IBM SPSS software (version 22.0; IBM). Quantitative parameters were evaluated for descriptive analysis by calculating the mean, standard deviation, and range. Continuous variables that followed a normal distribution were compared using an unpaired Student *t*-test. Radiation dose exposure was expressed as the DLP, measured in mGy·cm. To assess whether the HU values at monoenergetic 70 keV PCCT imaging associated with lytic bone lesions were significantly different from those associated with sclerotic bone lesions, an independent (unpaired) *t*-test was employed. RStudio (2023.06.0) was used to determine the correlation coefficient between lesion morphology (binary coding: sclerotic = 0; lytic = 1) and monoenergetic 70-keV attenuation values. Statistical significance was defined as *p* < 0.05. The primary endpoint was the feasibility of performing PCCT-guided bone biopsy with real-time BME mapping. Secondary endpoints included diagnostic yield, diagnostic accuracy, radiation dose parameters, and procedural time. An a priori feasibility criterion was established and defined as successful completion of the biopsy without requiring conversion to an alternative imaging guidance technique.

The final diagnosis (reference standard) was established using a predefined hierarchy: histopathology or microbiological confirmation when available; clinical and radiologic follow-up at ≥ 3 months for non-diagnostic or discordant biopsy results. A positive diagnostic yield is defined as successfully obtaining a definitive histological diagnosis from the biopsy specimens submitted. In contrast, a non-diagnostic yield refers to the failure to achieve a conclusive histological diagnosis. Diagnostic accuracy was measured by the level of agreement between the biopsy results and the final diagnosis and was calculated against this reference standard. A 2 × 2 contingency table (true positives, false positives, true negatives, false negatives) was used, and exact 95% confidence intervals were obtained using Wilson’s method.

## Results

**Fig. 1:**
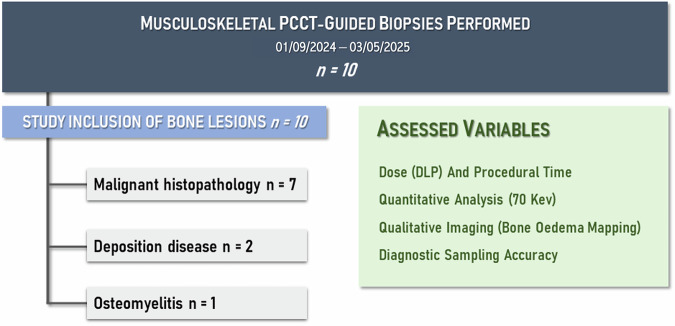
Study the inclusion process of PCCT-guided bone biopsies with exclusion criteria. DLP, Dose-length product; PCCT, Photon-counting computed tomography

A total of ten patients referred for a PCCT-guided bone biopsy, aged 60.5 ± 13.5 years (mean ± standard deviation), five women and five men, were enroled in the study (Fig. 1). Each patient contributed a single target lesion to the analysis; therefore, diagnostic yield, accuracy, and morphologic classification (lytic *versus* sclerotic) were assessed per patient.

The overall diagnostic yield for all bone lesions examined in this study stood at 70% (7/10), with an overall diagnostic accuracy of 87.5% (7/8).

There were 7 tumour bone metastases, 1 bone osteomyelitis, and 2 bone marrow infiltrative disease (Table 2). A per-patient reference standard was available in all cases: histopathology in eight patients and clinical/radiologic follow-up at ≥ 3 months in two patients. One nondiagnostic biopsy corresponded to a lesion subsequently confirmed as metastatic on follow-up (false negative), whereas another nondiagnostic biopsy remained stable on imaging, constituting a true negative. The corresponding 2 × 2 contingency table and 95% confidence intervals for diagnostic yield and accuracy are provided in the Supplementary material.

**Table 2:** Per-lesion demographic and clinical data

Age (years)	60.5 ± 13.5
Sex	
Male	5 (50%)
Female	5 (50%)
Location	
Axial	5 (50%)
Appendicular	5 (50%)
Bone matrix	
Lytic	5 (50%)
Sclerotic	5 (50%)
Procedural average time (min)	48.6 ± 8.6
DLP (mGy·cm)	644.5 ± 112.1
Final diagnosis	
Malignant disease	7 (70%)
Osteomyelitis	1 (10%)
Deposition disease	2 (20%)

Data are given as mean ± standard deviation or absolute values (percentage)

*DLP Dose*-length product

The mean operation time was 48.6 ± 8.6 min, ranging from 40 to 66 min per bone biopsy procedure.

A mean DLP of 644.5 ± 112.1 mGy·cm was found for PCCT-guided bone biopsy. The mean DLP for lytic lesions was 614.6 mGy·cm, while for sclerotic lesions, it was 674.4 mGy·cm.

### Quantitative imaging: monoenergetic 70 KeV

**Fig. 2:**
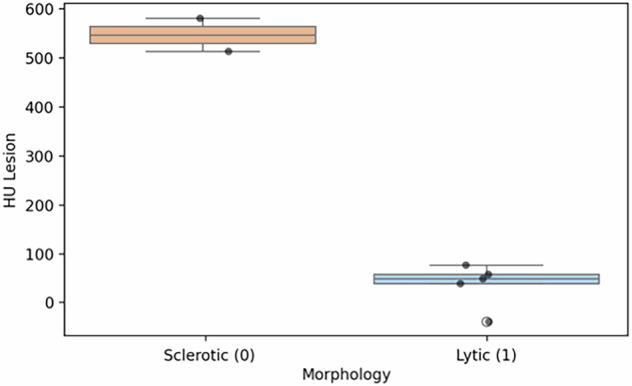
Box-plot distribution of CT attenuation values by lesion morphology. Sclerotic lesions (coded 0; *n* = 5) clustered at much higher HU values, reflecting denser mineralised bone matrix. Conversely, lytic lesions (coded 1; *n* = 5) exhibit lower HU values, consistent with bone matrix destruction. The near-complete separation between the two groups (*r* = -0.84; *p* = 0.002). HU, Hounsfield Units

The *t*-test revealed a statistically significant difference between lytic and sclerotic lesions (*p* = 0.009): sclerotic lesions exhibited markedly higher values (mean 476.2 HU) compared with lytic lesions (mean 42.6 HU). The negative *t*-statistic (-4.55) indicates that the mean monoenergetic HU value for lytic lesions is significantly lower than that for sclerotic lesions. A strong negative correlation was observed between lesion morphology (binary coding: sclerotic = 0; lytic = 1) and monoenergetic 70-keV attenuation values (*r* = -0.84; *p* = 0.002) (Fig. 2).

### Qualitative imaging: bone marrow colour map

**Fig. 3:**
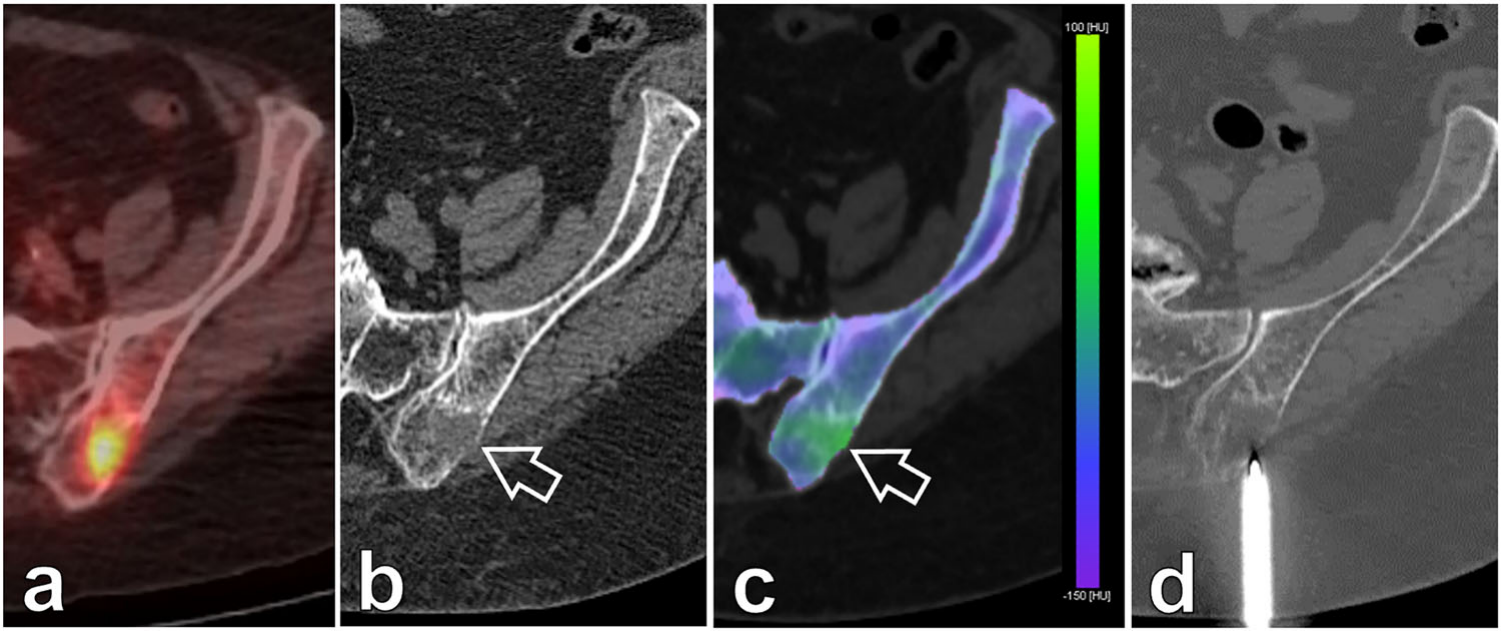
An 89-year-old female patient with a history of stage IV breast cancer was referred for biopsy of a suspicious lesion after a follow-up positron emission tomography/CT (**a**). **b** Monoenergetic axial 70-keV bone algorithm reconstruction (0.6 mm thickness; Br80) PCCT-guided biopsy image confirmed the presence of a suspicious lesion (arrow) with a heterogeneous, ill-defined lytic matrix located within the posterior aspect of the left iliac blade. **c** Real-time BME mapping (2-mm thickness; Qr40) depicted increased and pathological values within the lesion, colour-coded in shades of yellow/green (arrow), while healthy marrow values are colour-coded in blue/violet as seen in the remote iliac bone. **d** Allowing for an accurate and precise intraprocedural bone biopsy guidance of the target oedematous region with a drilling system. Final diagnosis was breast metastatic disease. BME, Bone marrow oedema; PCCT, Photon-counting computed tomography

**Fig. 4:**
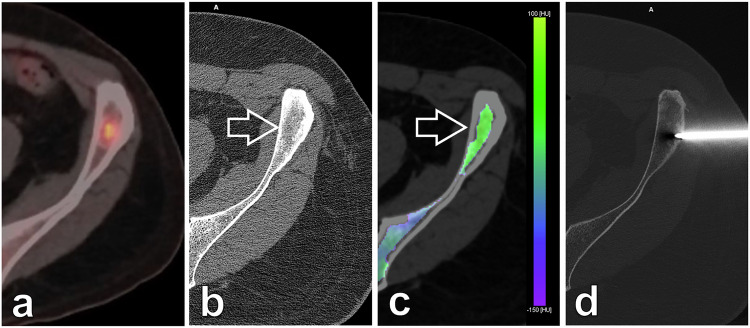
A 47-year-old male patient with a history of locally advanced adenoid cystic carcinoma showed an abnormal uptake focus in the left iliac blade on a positron emission tomography/CT follow-up scan (**a**) for which a bone biopsy was requested. **b** Monoenergetic axial 70-keV bone algorithm reconstruction (0.6-mm thickness; Br80) PCCT-guided biopsy image demonstrated a subtle sclerotic area (arrow) in the left iliac blade with no apparent identifiable bone lesion. **c** Real-time BME mapping (2-mm thickness; Qr40) depicted increased and pathological values, colour-coded in shades of yellow/green (arrow), while healthy marrow values are colour-coded in blue/violet as seen in the remote bone. In cases where a lesion detected by other imaging techniques does not translate on CT, real-time BME mapping allows for a confident intraprocedural bone biopsy guidance (**d**) towards the pathologic region of interest, documenting that the obtained samples followed the desired trajectory. Final diagnosis was metastatic disease. BME, Bone marrow oedema; PCCT, Photon-counting computed tomography

**Fig. 5:**
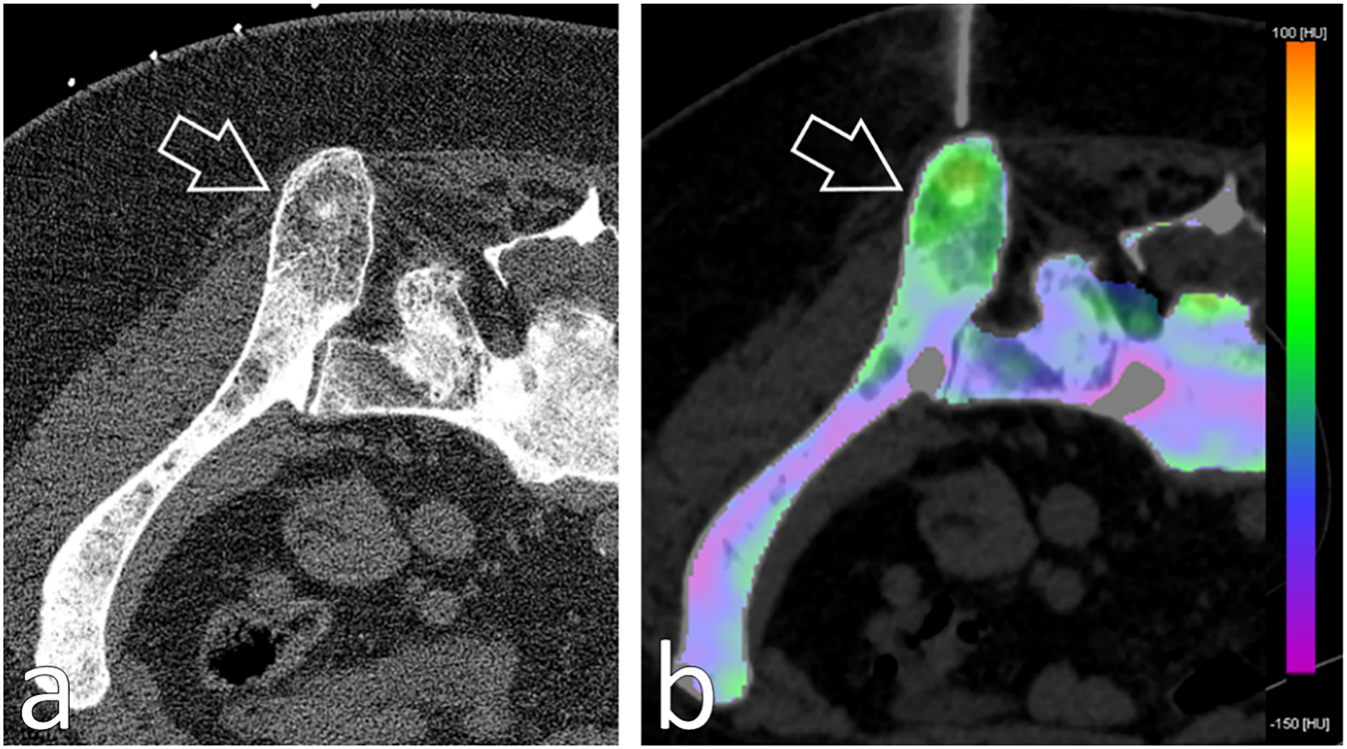
An 81-year-old patient with a history of locally advanced infiltrating ductal carcinoma treated by surgery and chemotherapy reported a new onset of pain during follow-up. **a** Monoenergetic axial 70-keV bone algorithm reconstruction (0.6-mm thickness; Br80) PCCT-guided biopsy image showed diffuse changes in trabecular bone microarchitecture with ill-defined lytic and sclerotic areas distributed throughout the pelvis, for which a bone biopsy was requested. **b** Real-time BME mapping (2-mm thickness; Qr40) displayed increased values (arrow), colour-coded in shades of yellow/green (arrow), which translates as a pathologic area; meanwhile, healthy marrow values are colour-coded in blue/violet as seen in the remote bone. In cases of mixed lesions, intraprocedural real-time BME mapping enables precise targeting of the region of interest, providing accurate guidance and confident sampling, thereby enhancing diagnostic yield. Final diagnosis was breast metastatic disease. BME, Bone marrow oedema; PCCT, Photon-counting computed tomography

Evaluating BME through the real-time three-material decomposition of VNCa reconstructions allowed us to accurately assess the region of interest for the bone biopsy needle. Examples of lytic and sclerotic bone lesions are shown (Figs. 3–5).

## Discussion

In this study, we highlighted the potential of PCCT as a guiding technique for bone biopsy procedures, demonstrating its ability to reduce radiation exposure significantly. Specifically, from our study, PCCT achieved an average DLP of 644.5 mGy·cm for CT-guided bone biopsy, which is lower compared to the reported DLP value of 733 mGy·cm using conventional CT scans [14] and 784 mGy·cm with dual-energy CT techniques [15, 16]. In this cohort, all metrics were evaluated per patient to avoid spectrum inflation from multiple lesions in the same individual and to provide a clearer interpretation of targeting performance.

PCCT represents a significant advancement in imaging technology, offering unique features that improve procedural accuracy and efficiency [11]. Similar to dual-energy CT, PCCT utilises spectral imaging capabilities to differentiate materials based on their atomic number and electron density. This enables the subtraction of calcium from trabecular bone, facilitating the identification of BME [17]. Such imaging provides diagnostic information comparable to magnetic resonance imaging, particularly T2-weighted fat-suppressed images, and is highly effective in detecting BME in pathological conditions such as neoplasms [18].

Notably, PCCT enables real-time imaging of intralesional bone oedema during biopsy procedures, facilitating precise localisation of the area of interest. This capability allows for dynamic guidance of the biopsy system as it advances toward the targeted region, enhancing the accuracy of the procedure, particularly in cases where precise lesion targeting is paramount.

In addition to improving accuracy, PCCT positively impacts procedural efficiency and workflow. By enabling real-time visualisation of bone oedema, PCCT reduces the need for repeated scans and adjustments, therefore streamlining the process and minimising procedural time. In fact, the operation time was 49 min, comparable to previous reports [19]. This not only improves patient comfort and reduces radiation exposure but also optimises clinical workflow, allowing for a greater number of procedures to be performed within a given timeframe.

Previous studies have noted that conventional CT-guided biopsies often exhibit lower diagnostic yield and a significant false-negative rate [20], particularly in sclerotic lesions [16]. These limitations frequently arise from the inability of conventional CT to reliably distinguish between viable tumour tissue and reactive sclerosis. In contrast, this study has shown promising results for monoenergetic 70 keV imaging in PCCT, with a statistically significant difference observed between lytic and sclerotic lesions (*p* = 0.009). Furthermore, a strong negative correlation (*r* = -0.84; *p* = 0.002) was found between lesion morphology (binary coding: sclerotic = 0; lytic = 1) and monoenergetic 70-keV attenuation values. These findings underscore the potential of monoenergetic 70 keV imaging to enhance the differentiation between lytic and sclerotic lesions, thereby improving diagnostic accuracy and visualisation of bone matrix. In the present study, diagnostic yield reflects only the proportion of biopsies providing a definitive histopathologic diagnosis, whereas diagnostic accuracy reflects agreement with the per-patient reference standard. Importantly, final diagnoses in nondiagnostic cases were established through structured clinical and radiologic follow-up, preventing misclassification. Although the pathologist was not formally blinded to clinical information, biopsy interpretation was performed independently, which limits verification bias.

Additionally, PCCT improves the visualisation of trabecular bone microarchitecture and enhances diagnostic precision, particularly in identifying and characterising lytic lesions, with slice thicknesses as thin as 0.2 mm, surpassing the capabilities of conventional energy integrating detector CT systems, which are constrained by larger detector elements and higher noise levels [21]. This improved resolution is particularly beneficial for identifying small or early-stage lytic lesions [10], which are often challenging to detect with conventional imaging methods [22]. It also facilitates more accurate targeting during procedures such as bone biopsies, thereby reducing the risk of sampling errors and improving diagnostic reliability.

This study has several limitations that should be acknowledged. First, it is a single-centre study with a limited number of patients, which restricts the ability to conduct a comprehensive analysis of subgroups, particularly in assessing lytic and sclerotic bone lesions. Second, the qualitative analysis of real-time bone oedema is inherently subjective and may vary between different observers, potentially affecting the consistency of the findings. Third, when a bone lesion is located outside the centre of the CT scan, repeated preliminary acquisitions are often necessary to ensure the safety and accuracy of the multi-energy information collected. Fourth, the reconstruction settings for PCCT, including resolution, kernel, denoising, and radiation dose, can vary depending on the affected body part, potentially influencing diagnostic imaging features. Additionally, the long-term clinical outcomes associated with PCCT-guided bone biopsies, particularly regarding their impact on patient management and overall prognosis, warrant further exploration in future research.

Despite these limitations, the overall findings highlight the clinical potential of this technique. PCCT-guided bone biopsy with real-time BME mapping was feasible and provided promising guidance capabilities in this small exploratory cohort. The technique enabled improved visualisation of intramedullary changes and yielded encouraging diagnostic performance metrics. While preliminary, these results support further evaluation in larger, controlled studies to better define its clinical value and comparative effectiveness.

## **Additional file 1: Table S1**. Per-procedure DLP table. **Table S2**. Per-patient biopsy result, reference standard, and diagnostic classification. **Table S3**. Contingency table for diagnostic accuracy (n = 10). **Table S4**. Diagnostic yield and diagnostic accuracy with 95% confidence intervals (CIs).

Supplementary information

## Data Availability

The datasets that support the findings of this study are available from the corresponding author upon reasonable request.

## Abbreviations

BME: Bone marrow oedema
PCCT: Photon-counting computed tomography
VNCa: Virtual non-calcium
