# Supplementary material for: Photon-counting CT-guided bone biopsy with real-time bone marrow edema mapping
Source: Eur Radiol Exp. 2026 Mar 16;10:28. doi: 10.1186/s41747-026-00690-6 (PMC12992892; doi:10.1186/s41747-026-00690-6)
Supplement: Supplementary file 1 — Supplementary information [file 41747_2026_690_MOESM1_ESM.pdf]

Photon-counting CT-guided bone biopsy with real-time bone marrow edema mapping.

ELECTRONIC SUPPLEMENTARY MATERIAL

Table S1. Per-procedure DLP table. *DLP*: Dose length product.

Group	n	Mean DLP (mGy·cm) ± SD
Lytic	5	614.6 ± 115.5
Sclerotic	5	674.4 ± 112.6
Overall	10	644.5 ± 112.1

Table S2 Per-patient biopsy result, reference standard, and diagnostic classification

Patient	Biopsy Result	Final Diagnosis (Reference Standard)	Reference Standard Method	Concordant?	Classification
1	Positive	Metastasis from breast carcinoma	Histopathology	Yes	True positive
2	Positive	Metastasis from prostate carcinoma	Histopathology	Yes	True positive
3	Non-diagnostic	Metastasis from colorectal carcinoma	Clinical + radiologic progression (3-month follow-up)	No	False negative
4	Positive	Metastasis from breast carcinoma	Histopathology	Yes	True positive
5	Positive	Osteomyelitis	Histopathology (culture-positive)	Yes	True positive
6	Positive	Deposition of Gaucher disease	Histopathology	Yes	True positive
7	Positive	Deposition of histiocytosis disease	Histopathology	Yes	True positive
8	Non-diagnostic	No evidence of bone malignancy	Stable imaging at 3 months	Yes	True negative*
9	Positive	Metastatic from adenoid cystic carcinoma	Histopathology	Yes	True positive
10	Positive	Metastasis from prostate cancer	Histopathology	Yes	True positive

* Negative biopsy and remained negative at 3-month radiologic follow-up.

Table S3 Contingency table for diagnostic accuracy (n = 10).

	**Reference standard positive**	**Reference standard negative**	**Total**
Biopsy Positive	7 (true positive)	0 (false positive)	7
Biopsy Negative	1 (false negative)	1 (true negative)	2
Total	8	1	9*

*One non-diagnostic biopsy belonged to a reference-negative patient and was included as a true negative.

Table S4 Diagnostic yield and diagnostic accuracy with 95% confidence intervals (CIs)

**Metric**	**Percentage and ratio**	**95% CI (Wilson)**
Diagnostic yield	70% (7/10)	0.40–0.89
Diagnostic accuracy	87.5% (7/8)*	0.53–0.98

*Accuracy computed among cases with definitive reference standard (*n* = 8
